# Mitochondrial Dysfunction in Parkinson’s Disease: Focus on Mitochondrial DNA

**DOI:** 10.3390/biomedicines8120591

**Published:** 2020-12-10

**Authors:** Olga Buneeva, Valerii Fedchenko, Arthur Kopylov, Alexei Medvedev

**Affiliations:** Institute of Biomedical Chemistry, 10 Pogodinskaya Street, 119121 Moscow, Russia; olbuneeva@gmail.com (O.B.); valfed38@yandex.ru (V.F.); a.t.kopylov@gmail.com (A.K.)

**Keywords:** Parkinson’s disease, Parkinson’s disease models, mitochondrial dysfunction, mitochondrial DNA, proteins encoded by mitochondrial genes, proteomics

## Abstract

Mitochondria, the energy stations of the cell, are the only extranuclear organelles, containing their own (mitochondrial) DNA (mtDNA) and the protein synthesizing machinery. The location of mtDNA in close proximity to the oxidative phosphorylation system of the inner mitochondrial membrane, the main source of reactive oxygen species (ROS), is an important factor responsible for its much higher mutation rate than nuclear DNA. Being more vulnerable to damage than nuclear DNA, mtDNA accumulates mutations, crucial for the development of mitochondrial dysfunction playing a key role in the pathogenesis of various diseases. Good evidence exists that some mtDNA mutations are associated with increased risk of Parkinson’s disease (PD), the movement disorder resulted from the degenerative loss of dopaminergic neurons of *substantia nigra*. Although their direct impact on mitochondrial function/dysfunction needs further investigation, results of various studies performed using cells isolated from PD patients or their mitochondria (cybrids) suggest their functional importance. Studies involving mtDNA mutator mice also demonstrated the importance of mtDNA deletions, which could also originate from abnormalities induced by mutations in nuclear encoded proteins needed for mtDNA replication (e.g., polymerase γ). However, proteomic studies revealed only a few mitochondrial proteins encoded by mtDNA which were downregulated in various PD models. This suggests nuclear suppression of the mitochondrial defects, which obviously involve cross-talk between nuclear and mitochondrial genomes for maintenance of mitochondrial functioning.

## 1. Introduction

More than two centuries ago, James Parkinson described in his famous monograph “An Essay of the Shaking Palsy” (1817) the main clinical symptoms of one of the most widespread age-related neurodegenerative diseases now known as Parkinson’s disease (PD) [1,2]. Elucidation of the pathological basis for the appearance of these symptoms (degeneration of dopaminergic neurons of *substantia* *nigra pars compacta* accompanied by striatal dopamine depletion) took more than one century after that description [1,2]. The development of various experimental models started in the second half of the last century and the genetic analysis of PD patients revealed molecular mechanisms crucial for important aspects of various forms of PD (both sporadic and familial) [1,2,3].

Results of numerous studies point to an important role of mitochondria and mitochondrial dysfunction in the pathogenesis of PD, which started to be considered as a common feature or even as a cause of PD after discovery of MPTP (1-methyl-4-phenyl-1,2,3,6-tetrahydropyridine)-induced Parkinsonism [4], originating from the mitochondrial complex I inhibition and complex I deficiency recognized in *substantia nigra* of PD patients [5,6].

In addition to complex I defects found mainly in *substantia nigra* of aged and PD patients, deficient complex I activities have also been found in platelets, skeletal muscles [7,8,9,10], skin fibroblasts [11] from PD patients, but not in lymphocytes [10]. This suggests that the complex I defect seen in PD and aging is systemic and has “a genetic basis” [12]. The evaluation of molecular events leading to manifestation of PD revealed the involvement of mitochondrial proteins in the mechanisms of neuron damage and neurodegeneration. Being encoded by both nuclear and mitochondrial genomes, they have a significant impact on both mitochondrial function/dysfunction and also numerous interactions of mitochondria with other intracellular organelles. This review is focused on the particular role of mitochondrial DNA (mtDNA) and its changes associated with PD development and mitochondrial dysfunction.

## 2. Mitochondrial DNA: Structure, Functions, Mode of Replication and Transcription of Mitochondrial Genes

**Figure 1:**
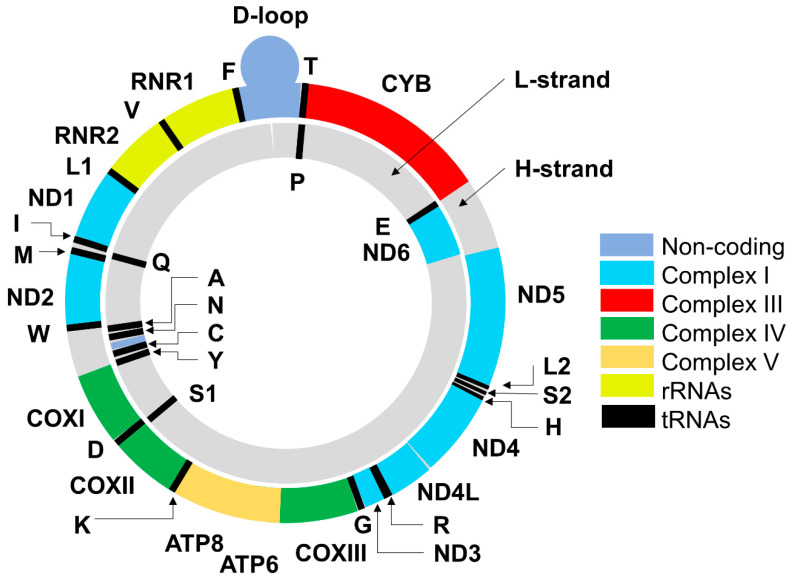
The scheme of the circular, double-stranded human mtDNA. The outer circle denotes the heavy (H) strand of the mtDNA and the inner circle denotes the light (L) strand. Colors denote genes encoding protein subunits of complexes I-V, ribosomal RNAs, transfer RNA (tRNAs designated using single-letter amino acid abbreviations), and a non-coding region (D-loop). Genes encoding protein subunits correspond to symbols of these subunits shown in Table 1. Origins of heavy and light strand replications are not shown.

Mitochondria, the energy stations of the cell, are the only extranuclear organelles, containing their own (mitochondrial) DNA (mtDNA), originally discovered in 1963 [13,14], and the protein synthesizing machinery [15,16]. The mammalian mitochondrial genome is a multicopy circular, double-stranded DNA of about 16.5 kilobases in length; it encodes two ribosomal RNAs, 22 transfer RNAs, and just 13 protein subunits of the electron transport chain [17,18,19,20] (Table 1, Figure 1).

MtDNA is maternally inherited. Many researchers are convinced that paternal mtDNA does not enter into the fertilized oocyte during fertilization [26,27], others suggest that sperm mitochondria are selectively targeted for degradation after fertilization [28,29,30]. In any case, the dogma that in humans mtDNA is maternally inherited, is used in genetic consultations [31]. The healthy cell contains several thousand mtDNAs, each of which has an identical nucleotide sequence (homoplasmy). Aging and diseases associated with mitochondrial dysfunction are characterized by the accumulation of somatic mutations and coexistence of wild type and mutant mtDNAs (heteroplasmy) and there is a correlation between the percent of mutant mtDNAs and manifestation of the disease [32].

The light (L) and heavy (H) strands of mtDNA differ in their base composition [33]: The L-strand is rich in cytosines, while the H-strand is rich in guanines. The L-strand contains eight genes of tRNA and the gene of NADH-ubiquinone oxidoreductase chain 6 protein (*ND6*). Twenty eight other genes are located on the H-strand. They encode 14 tRNAs, two rRNAs (12S and 16S rRNA), and 12 polypeptides involved in electron transport and oxidative phosphorylation: Six subunits of the NADH-ubiquinone oxidoreductase complex (ND1, ND2, ND3, ND4, ND4L, ND5), one subunit (cytochrome *b*; CYB) of the cytochrome *bc*_1_ complex, three subunits of the cytochrome *c* oxidase complex (COXI, COXII, COXIII), and two subunits of the ATP synthase complex (ATP 6 and ATP8) (Figure 1).

MtDNA contains a noncoding region also known as the control region where promoters for polycistronic transcription (one for each mtDNA strand) are localized: The light strand promoter (LSP) and the heavy strand promoter (HSP) [34,35]. The control region also contains the origin for heavy strand DNA replication (OH), while the origin for light strand replication (OL) is located in the coding region, within a tRNA cluster [34,36]. The human and mammalian mitochondrial genome has its specific features different from the universal genetic code. Human and mammalian mtDNA contains 64 codons and four of them are STOP codons. Codon AUA of human mitochondria encodes methionine rather than leucine in the standard code. Arginine codons AGA and AGG are STOP codons in mtDNA. UGA, one of the three STOP codons of the standard code, encodes tryptophane in mtDNA. In addition, a single tRNA molecule of mtDNA can identify all codons of a four-codon family. This explains, why only 22 tRNAs are sufficient for identifying all the 64 codons, whereas generally there should be not less than 32 tRNAs [37]. Another feature typical of mtDNA is the high mutation frequency, which may be explained by the effects of reactive oxygen species (ROS) attributed to the proximity of the mitochondrial electron transport chain.

A short three-strand structure of mtDNA, known as the D-loop (D for Displacement) forms due to the DNA replication and is situated in its non-coding region. It participates in the regulation of mtDNA replication and transcription, which involves mitochondrial proteins encoded by the nuclear genome; they are synthesized outside mitochondria and then transported to these organelles. Human mtDNA is very compact and it lacks introns. Although the mitochondrial genome encodes some basic components required for mRNA translation, the process of protein biosynthesis strictly depends on the cell nucleus, as enzymes and other protein factors needed for replication, repair, transcription, and translation are encoded by nuclear genes. In the context of neurodegenerative disorders associated with DNA damage, this suggests the possibility of DNA damage in both nuclear DNA and mtDNA.

MtDNA is located in the mitochondrial matrix as the nucleoprotein complexes, nucleoids [38,39]. The macromolecular structure of the nucleoid includes a central zone containing mtDNA and proteins needed for replication and transcription, and a peripheral zone, formed by factors involved in the association with mitochondrial ribosome, communication, and signaling with other subcellular compartments [38]. Proteomic studies employing various approaches, including nucleoid cross-linking, immunoprecipitation of known nucleoid proteins, and proximity-based labeling methods, revealed a representative group of mtDNA binding proteins [40,41,42,43,44,45,46,47]. Despite significant variations in the number of identified mtDNA binding proteins and critical evaluation of the methodical approaches used by different groups (e.g., [39,46]), these results indicate a rather tight association of nucleoids with the inner mitochondrial membrane, where the major sources of reactive oxygen species (ROS) are located. Indeed, electron microscopy studies have shown that nucleoids interact with the inner mitochondrial membrane [48]. Very recent electron microscopy data suggest that the mitochondrial inner membrane protein complex MICOS (mitochondrial cristae organizing system) links nucleoids to the Miro1 protein (mitochondrial Rho GTPase 1) for their active transportation to the peripheral zone of the cell [49]. This obviously explains detection (as contaminants) of NADH dehydrogenase subunits (complex I) and other proteins such as ATP synthase subunits, adenine nucleotide translocator 3, voltage-dependent anion-selective channel protein 3 (*VDAC3*), etc. (e.g., [42]). Since the mitochondrial nucleoid lacks a membrane, it is considered as a nucleoprotein complex [46]. Recently, a group of mitochondrial histones has been identified [43], however, their anchoring to the outer mitochondrial membrane [11] indicates that they are not involved in the interaction with mtDNA. Therefore, although the mtDNA package into nucleoids provides some protection of the mitochondrial genome, it is highly vulnerable to various damaging effects.

Studies based on electron microscopy and two-dimensional electrophoresis data revealed specific features of mammalian mtDNA replication. Three different models were suggested to explain these data. According to the strand displacement model, mtDNA replication is unidirectional, asymmetric, and asynchronous [50,51,52,53]. It begins in the origin of replication of heavy strand (OH) and while the synthesis of a new H strand takes place, the parental H-strand is covered with a single strand binding (SSB) protein. When the replication fork reaches OL, DNA forms a stem-loop structure, thus preventing SSB binding in this region. Then, RNA-polymerase POLRMT synthesizes a primer of about 25 nucleotides in length at the single strand region of the loop, and DNA polymerase gamma (POLG) continues the DNA synthesis. According to the strand-coupled model, the mtDNA replication initiates bidirectionally from a broad region of several kilobases, including the gene-encoding region of mtDNA (Ori z) [36]. The OH region functions as a replication fork barrier. The synthesis of the leading and the lagging strands (both composed of DNA) proceeds synchronously. However, molecular mechanisms responsible for this type of replication have not been characterized [34]. The mtDNA replication model RITOLS (RNA incorporated throughout the lagging strand) explains the experimental data of complementary RNA molecules discovered only in the L strand of DNA. Later, this model was complemented and renamed as a bootlace model (or bootlace strand-asynchronous replication). According to this model, RNA transcripts synthesized on the lagging strand are incorporated in the intermediates of replication, as the replication fork moves forward, and then these transcripts are replaced by the DNA lagging strand. The bootlace strand-asynchronous replication model suggests that replication initiates with the synthesis of the H strand at one of two sites, OH or Ori-b. The leading strand (H strand) synthesis proceeds unidirectionally with the simultaneous incorporation of RNA fragments into the lagging strand. The RNA lagging strand induces hybridization of mitochondrial transcripts to the parental H strand. The initiation of the synthesis of the lagging L strand can begin at several origins, not exclusively at OL. It proceeds unidirectionally, while RNA lagging strands are gradually removed. A lack of consensus on a common mechanism suggests the existence of several modes of the mtDNA replication. By analogy with the replication-transcription switch in human mitochondria [54], it is reasonable to suggest that there are regulatory factors switching these replication modes in dependence of various cell conditions.

Mitochondrial transcription and subsequent RNA processing are carried out by specialized nuclear-encoded proteins. The transcription of human mitochondrial genes involves DNA-dependent RNA polymerase POLRMT [55], which interacts with mitochondrial transcription factor A (TFAM) and mitochondrial transcription factor B2 (TFB2M). TFAM is a DNA-binding protein, which plays an important role not only in transcription activation, but also in mtDNA package within the nucleoid [46]. TFB2M is essential for promoter melting during the initiation of transcription [56]. Although only these two transcription factors are needed for transcription of the mitochondrial genes in vitro [57], animal experiments performed using manipulations with the recently discovered mitochondrial transcription elongation factor (TEFM) suggest its role in the regulation of both transcription elongation and RNA processing [58]. Transcription from the heavy (HSP) and light (LSP) strand promoters yields long polycistronic transcripts. Endonuclease excision of mitochondrial tRNAs separating coding sequences of mitochondrial rRNAs and protein coding sequences in the polycistronic transcripts is accompanied by the release of all types of mitochondrial RNAs (tRNAs, rRNA, and mRNA) [17,55]. After excision from the primary transcript, almost all mRNAs (except *MT-ND6* mRNA, the only protein-coding transcript encoded on the light strand), undergo 3′ polyadenylation. Polyadenylation of mitochondrial mRNAs is carried out by poly(A) RNA polymerase [59,60,61]. The stability of HSP-derived mitochondrial transcripts is controlled by the Leucine-rich pentatricopeptide repeat motif-containing protein (LRPPRC), which is necessary for polyadenylation [62,63]. The absence of LRPPRC has a significant impact on the steady-state levels of mRNAs but not rRNAs and tRNAs, and *LRPPRC* knockout mice are characterized by a loss in HSP-derived transcripts, loss of poly(A) tails, and a severe translational defect [62,63].

## 3. MtDNA Oxidation and Repair Mechanisms

Mitochondrial dysfunction can be induced by mtDNA damage including point mutations, large-scale deletions, and oxidation [64,65,66,67]. Convincing evidence exists that under conditions of oxidative stress mtDNA damage is more extensive and persists longer than damage of nuclear DNA [68]. MtDNA is especially vulnerable to oxidative damage, due to the nucleoid location in close proximity to the inner mitochondrial membrane where the oxidative phosphorylation (OXPHOS), the major source of cell reactive oxygen species (ROS) occurs [66,67,68]. In addition, mitochondria are highly enriched in iron [69], which favors the formation of aggressive ^•^OH radical, preferentially reacting with intramitochondrial proteins and mtDNA due to its short half-life [70].

All the nitrogenous bases of DNA adenine, guanine, cytosine, and thymine and their corresponding deoxynucleosides are highly susceptible to oxidative damage [66,67]. Although various oxidized base adducts can be potentially formed during the ROS attack on the DNA [71], guanine is the most readily oxidizable base [66,67]. In the context of DNA oxidation, 8-oxo-guanine (8-oxoG) is the most studied form of oxidized DNA bases. Cell exposure to hydrogen peroxide is accompanied by a continuous accumulation of 8-oxoG both in nuclear DNA and mtDNA [72]. Such point lesions may be repaired by the base excision repair (BER) system [73]. BER represents the primary nuclear and mitochondrial repair pathway for oxidative DNA damage. It is initiated by DNA glycosylase, which is responsible for recognition and removal of the damaged base; the resultant abasic site is then processed by a short-patch repair or long-patch repair employing different proteins to complete BER [73]. Oxidized bases are generally removed by so-called bifunctional DNA glycosylases. In the context of mtDNA, 8-oxoguanine DNA glycosylase (OGG1) is especially important. It has two isoforms, one of which is located in the nucleus and mitochondria, while the other one is located in the mitochondria [74]. Hepatic mitDNA isolated from Ogg1-null mutant mice had a much higher (more than 20-fold) level of 8-oxoG than wild-type C57Bl/6J mice [75]. This points to the very important role of this enzyme for 8-oxoG elimination. Another enzyme that suppresses accumulation of 8-oxoG both in nuclear and mitochondrial DNA is MTH1 (MutT homolog protein 1). This oxidized purine nucleoside triphosphatase can hydrolyze oxidized purine nucleoside triphosphates (8-oxo-dGTP, 2-hyrdoxy-dATP) to the corresponding monophosphates [66,72]. MTH1-null mice were characterized by a higher accumulation of 8-oxoG in mtDNA but not in nuclear DNA. This was accompanied by a more significant decrease in tyrosine hydroxylase and dopamine transporter immunoreactivities than in wild-type mice [66,72,76]. Abasic sites, formed in mtDNA due to a spontaneous base loss or induced by ROS, are also harmful particularly in mitochondria due to the decreased activity of mitochondrial DNA polymerase [77]. Generation of a mouse model with a mutated version of the uracil N-glycosylase (UNG) DNA repair enzyme, which removed thymine from the mitochondrial genome, revealed highly elevated levels of apyrimidinic sites in hippocampal mtDNA [78].

Studies performed using neuroblastoma SH-SY5Y cells treated with hydrogen peroxide (100–1000 µM) revealed that at lower H_2_O_2_ concentrations the non-coding regulatory D-loop was more vulnerable to H_2_O_2_ induced mtDNA damage than the three coding regions tested. At a higher concentration of H_2_O_2_ (750–1000 µM) the difference in the lesion rates disappeared [79]. Since replication of mtDNA starts in the D-loop region [34,35,36] its higher sensitivity to ROS (H_2_O_2_) suggests different rates of mtDNA damage and copy number recovery. Indeed, the initial hydrogen-peroxide mtDNA damage almost reversed after 48 h, while the mtDNA copy number was reduced to 50% in the SH-SY5Y cells [79].

## 4. Age-Related Changes in mtDNA

Generally, age-associated oxidation of mtDNA originates from an increased oxidative attack to the nucleic acids and a decreased efficacy in mtDNA repair mechanisms [67,80,81]. The aging brain is characterized by an increased oxidative damage of mtDNA evaluated by the formation of 8-oxoG, commonly considered as the marker of oxidative DNA damage [80,81]. The age-related increase in 8-oxoG was found in several cerebral regions of aged human brains [82]. The extent of 8-oxoG in mtDNA was one order of magnitude higher than in nuclear DNA [82]. The same trend was also detected in brains (and hearts) of aged mice, rats, guinea pigs, and rabbits and also sheep, pigs, cows, and horses [83]. Moreover, oxidative damage of mtDNA inversely correlated with the maximum life span of these animals. This suggests the applicability of animal models for the study of PD.

The age-related increase in oxidative mtDNA damage is obviously associated with age-related impairments of the components of the mitochondrial BER machinery (OGG1, UDG, APE1, and polymerase γ) [84,85,86]. Mouse brain mtDNA repair activities demonstrated clear regiospecific differences [86]. For example, in mice mtDNA glycosylase activities were lower in hippocampal than in cortical mitochondria. Mitochondrial AP endonuclease activity increased in old animals in both brain regions, while cortical but not hippocampal mtDNA glycosylase activities declined with age [86]. In *substantia nigra* of neurologically healthy individuals the number of hOGG1-2a-positive neurons (stained with antibody to the mitochondria specific isoform) demonstrated an age-dependent increase [87].

The analysis of blood samples (*n* = 2491, age-range 0–60 years) revealed a significant age-dependent increase in the mtDNA heteroplasmy [88]. These results are consistent with high levels of mtDNA deletions found in *substantia nigra* neurons of the aged brain [89,90]. Nevertheless, dopaminergic *substantia nigra* neurons of neurologically healthy individuals demonstrated an age-related increase in mtDNA copy number and maintained the pool of wild-type mtDNA population despite accumulated deletions [91].

Therefore, although it is reasonable to suggest that the increase in the oxidative damage of mtDNA could account for age-related accumulations of point mutations and deletions in the mitochondrial genome, there are certain concerns, whether these changes are a cause or consequence of the aging process [92].

## 5. MtDNA Changes in Parkinson’s Disease

### 5.1. MtDNA Mutations

Although mtDNA sequencing did not reveal characteristic pathogenic mutation(s) as a PD signature [93], there was an age-dependent increase in mtDNA deletions associated with respiratory chain dysfunction detected in the dopaminergic neurons of *substantia nigra* [89,90]. Dopaminergic neurons with normal cytochrome oxidase activity were characterized by very high levels of mtDNA deletions. The level of somatic mtDNA deletions was slightly higher in *substantia nigra* of PD patients than in age-matched controls. Since mtDNA deletions were not seen in other types of neurons in aged brains [89,90], it was suggested that they were specific for *substantia nigra* [89].

The other study also revealed that the number and variety of mtDNA deletions/rearrangements were selectively increased in postmortem samples of *substantia nigra* from PD patients as compared to both patients with other types of neurodegenerative pathology (movement disorders and Alzheimer’s disease) and aged controls [94]. Other brain regions of PD patients also demonstrated increased mtDNA deletions/rearrangements, thus indicating that in these patients mitochondrial dysfunction was not limited to the *substantia nigra* [94]. This suggests that the accumulation of mtDNA deletions/rearrangements could be not only a specific sign of PD but also an important factor responsible for the development of mitochondrial dysfunction and neurodegeneration in PD. Table 2 lists some mutations in mtDNA associated with PD.

Data in Table 2 show that not all mutations recognized in the mitochondrial genome are functionally linked to mitochondrial dysfunction and results of population genetic studies require further functional studies.

### 5.2. Substantia Nigra Samples from PD Patients

The analysis of 11 brain regions of postmortem brain samples from PD patients and controls revealed a selective increase in 8-oxoG levels in *substantia nigra* [124]. Since authors were limited in the biological materials they investigated total cell DNA. However, the accumulation of 8-oxoG was later demonstrated in mtDNA of nigrostriatal dopaminergic neurons of PD patients [66]. *Substantia nigra* neurons from aged individuals and PD patients also had high levels of deletions in mtDNA [89]. These mtDNA lesions were associated with respiratory chain deficiency.

Using ultradeep sequencing of mtDNA it has been shown that individual dopaminergic neurons of *substantia nigra* from PD patients contained a higher pool of mtDNA deletions than it was previously reported [125]. Each *substantia nigra* neuron contained more than 30 distinct mtDNA deletions and most of these deletions were found to be present in very low frequencies and were located in areas of perfect or interrupted homology [125]. In contrast to neurons of control individuals demonstrating an age-related increase in the mtDNA copy number and maintenance of wild-type mtDNA population, corresponding neurons from PD patients were characterized by depletion of the wild-type mtDNA population [91]. However, the neuronal mtDNA point mutational load did not increase in PD patients [91]. The other study revealed increased expression of the mitochondrial isoenzyme, OGG1, involved in mtDNA repair, in *substantia nigra* neurons of PD patients [87].

### 5.3. Other Cells from PD Patients

In addition to brain samples obtained *post mortem*, changes in mtDNA have been also investigated using biopsy samples from PD patients. The study of mtDNA replication and transcription in skin fibroblasts from patients with familial *LRRK2*-associated and idiopathic PD has shown that these cells obtained from both groups of patients are characterized by similar dysfunctions of the mtDNA replication and transcription machinery [126]. These included the accumulation of 7S mtDNA, low mtDNA replication, high heavy strand transcription, and low cell-free mtDNA release [126]. The latter (cell free mtDNA release) is currently considered as an active physiological process regulated by metabolic stress rather than a hallmark of cell lysis [126]. The altered level in 7S DNA, which plays a role in the switch between replication and transcription of mtDNA [54], is considered as a basic mechanism in the pathogenesis of idiopathic and monogenic *LRRK2*-associated PD.

### 5.4. Studies Using Cytoplasmic Hybrid (Cybrid) Cell Lines

In the context of studies aimed at elucidation of the role of mtDNA in mitochondrial dysfunction and pathogenesis of PD, the cytoplasmic hybrid studies performed using mitochondria from cells obtained from PD patients made a significant contribution [12,127,128]. Cybrid cells are generated by mixing contents of a non-nucleated cell with a nucleated cell. The nucleated cell is usually a tumor cell with depleted mtDNA (known as a ρ_0_ cell) and the platelet usually represents a source of the non-nucleated cell containing functionally competent mitochondria [129]. Since cybrids share the same nuclear genetic background, the differences in structure-functional parameters of the generated cybrid cells containing mtDNA from various sources are obviously originated from the differences in their mtDNA. Table 3 summarizes results obtained using such systems.

It appears that certain but not all studied cybrids containing mtDNA from PD patients differ from control cybrids containing mtDNA from non PD subjects. A decrease in Complex I activity and increased generation of ROS are consistent with results obtained using various cells from PD patients (see above). Since focal respiratory chain defects initially seen in platelets of patients with other pathologies (e.g., Alzheimer’s disease) or documented lesions of mtDNA (e.g., some mutations, see Table 2) have been reproduced in the cybrid cells, such cybrid cells represent an adequate model for characterization of molecular events associated with mitochondrial dysfunction. Other processes altered in cybrid cells, containing mitochondria from PD patients versus control cybrids (Table 3) reflect complex interactions, including both effects of components transferred together with mitochondria to the nucleated tumor ρ_0_ cell, as well as a functional crosstalk between mtDNA and nuclear DNA [140].

## 6. Studies Using Animal Models of Parkinson’s Disease

### 6.1. MPTP-Induced Parkinsonism

In the context of PD models in animals, MPTP-induced Parkinsonism is one of the commonly used experimental models reproducing the main neuropathological hallmarks of this disease [2,31,66,141,142]. In Parkinsonism induced by administration of MPTP, 1-methyl-4-phenyl-1,2,3,6-tetrahydropyridine (a protoxin) undergoes catalytic conversion by monoamine oxidase B (MAO B), which is self-inactivated during this process. The resultant neurotoxin MPP^+^ (1-methyl-4-phenylpyridinium) inhibits complex I of the respiratory chain and causes development of symptoms typical of PD [2,141,142].

A single dose administration of MTPT to mice (30 mg/kg) caused characteristic locomotor impairments, rapidly developed within 90–120 min [143,144]. They were accompanied by increased ubiquitination of oxidized proteins associated with brain mitochondria [144]. This points to an early involvement of mitochondria in the cell response to the administered toxin. Administration of the same dose of MPTP to mice caused accumulation of 8-oxoG in *striatum* and *substantia nigra* observed 12–24 h after the MPTP injection [66,72,76]. The accumulation of 8-oxoG in mtDNA induced by MPTP administration was more pronounced in MTH1-null mice defective in the *MTH1* gene encoding 8-oxo-dGTPase than in wild-type mice [66,76]. These changes (8-oxoG accumulation in mtDNA) appeared in *substantia nigra* and *striatum* prior to loss of their neurons [76]. Interestingly, the level of 8-oxoG in nuclear DNA insignificantly differed between wild-type and MTH1-null mice [66,76].

The treatment of mice with MPTP (10 mg/kg for 4 days with a 3-day interval after the last injection) caused mtDNA damage evaluated by quantitative PCR [145]. This damage was more pronounced in old (1-year old) than in young (22-day old) mice and *substantia nigra* DNA was more affected than *caudate-putamen* and *cerebellum* [145]. In vitro treatment of cells with MPP^+^, the toxin formed during MAO B-dependent conversion of MPTP, also caused mtDNA damage. For example, the treatment of SH-SY5Y cells with 1 mM MPP^+^ increased mitochondrial 8-oxoG after 1 h, while in the nucleus 8-oxoG accumulation was observed 2 h later [146].

However, despite early mtDNA damage observed within 12–24 h after MPTP administration (and development of movement disorder), proteomic analyses performed in other laboratories did not reveal significant alterations in the level of proteins encoded by mtDNA [147,148]. About two hundred genes, including genes encoding five subunits of complex I (*Ndufa10*, *Ndufb5*, *Ndufs2*, *Ndufs7*, *Ndufb9*) and several ATP synthase subunits responded to MPTP administration. However, the only significant change (downregulation) in the mitochondrial genome was found in the case of ATP synthase subunit 8 [147] encoded by the mitochondrial gene located on the H-strand (see Figure 1). This suggests preferential involvement of the nuclearly encoded mitochondrial proteins in the MPTP-induced changes at least within the particular protocol used: Four sequential injections of MPTP (15 mg/kg per injection) at 2 h intervals with bilateral removal of *striatum* and other brain regions (cortex, cerebellum, and the rest of the brain) 7 days after the injections [147]. We suggest that the selection of appropriate experimental protocols appears to play an important role especially if we take into consideration that the mtDNA damage and mtDNA copy number demonstrated different recoveries after the ROS-dependent treatment of cells (see the last paragraph of Section 3).

### 6.2. Rotenone-Induced Parkinsonism

The other popular toxin-based model of PD is rotenone-induced Parkinsonism. In this model, repeated systemic injections of the pesticide rotenone to rats caused the inhibition of mitochondrial complex I in the nigrostriatal dopamine system [149,150] and also in the mitochondria of peripheral organs (e.g., liver, [151]). Although histopathological and other examinations of about 40 organs revealed several crucial targets (liver, bone marrow, and bone) [151], the brain rotenone treatment caused selective degeneration of the nigrostriatal dopamine system and reproduced major clinical symptoms typical of PD [149,150]. In the rotenone model of PD in rats the mtDNA damage (apurinic/apyrimidinic (abasic) sites) was detected even after a single dose administration [150], which did not cause behavioral symptoms of Parkinsonism [149]. This mtDNA damage was detectable in *substantia nigra* (but not in the cortex) and occurred before signs of nigrostriatal system degeneration.

Recently, the performed proteomic analysis using IMR-32 cells treated with retinoic acid for their differentiation into dopaminergic neuron population has shown that the treatment with rotenone affected the expression of more than 400 proteins [152]. However, the only protein encoded by mtDNA was the COXII subunit and its level insignificantly differed from the control (the rotenone/control ratio was 1.06) [152]. It would be interesting to repeat such proteomic analysis using rotenone-treated animals, exhibiting symptoms of PD.

### 6.3. Polg Mutator Mice

DNA polymerase-γ is the principal enzyme in mtDNA replication and mtDNA proofreading. The lack of this enzyme causes early embryonic death [153], while a missense mutation causing amino acid substitution D257A only reduces the 3′–5′ exonuclease activity needed for proofreading without a significant change of the mtDNA replication capacity [154,155]. The homozygous *Polg^D257A/D257A^* mice carrying this mutation die prematurely at the age of about 12 months (41–59 weeks) due to increased random accumulations of mtDNA mutations, while wild-type mice live more than 2 years [154,155]. The knock-in mice developed an mtDNA mutator phenotype with a several fold increase in the levels of point mutations and increased amounts of deleted mtDNA. The rate of mtDNA mutations markedly differed in various tissues of the mitochondrial mutator mice [156].

In the skeletal muscle mitochondria of 11-month old *Polg* mutator mice, the total content of mitochondrial electron transport chain decreased by 35% (complex I); 37% (complex III), and by 50% (complex IV). The analysis of selected subunits revealed a significant decrease in both nuclear-encoded (NDUFA9 and NDUFS3 subunits of complex I, 29 kDa and 48 kDa subunits of complex III), and mitochondrial-encoded subunits (COXI subunit of Complex IV) [157]. Gene expression profiling revealed 97 differentially expressed genes with the highest upregulation of genes encoding extramitochondrial proteins (HD domain containing protein 3–9.7 fold; acetyl-CoA synthetase 6.8-fold; Cd209e gene-like protein E—4.1-fold). The most prominent downregulation was observed for alpha kinase 3 (−4.5-fold), immunoglobulin heavy locus (−4-fold), and 3-hydroxybutyrate dehydrogenase 1 (−3.6-fold) [157]. Interestingly, a similar study performed in young (3-month old) mutator mice did not reveal differentially expressed genes [157]. No changes were also observed in 8-oxodG levels in mtDNA between wild-type and mtDNA mutator mice [158].

Young (2–3 month old) *Polg^D257A/D257A^* mutator mice did not demonstrate higher neuronal vulnerability than the wild-type mice [159]. In addition, another study has shown that *DJ-1*-deficient mice, *Polg* mutator mice, and *DJ-1*-deficient *Polg* mutator mice had intact nigrastriatal pathways [160]. Neurons and muscle cells of mtDNA mutator mice maintained a well-preserved mitochondrial respiratory chain [161]. Mitochondrial levels of hydrogen peroxide in the studied tissues (liver, kidney, heart, and skeletal muscles) were basically the same in young *Polg* mutator mice and wild-type mice, while in mature mtDNA mutator animals were higher in the heart and kidney [162].

Nevertheless, quantitative proteomic profiling revealed brain proteins differentially expressed in the *Polg* mutator mice as compared with the wild-type mice [163]. At least several mitochondrial respiratory chain subunits were significantly decreased and these included cytochrome *c*-oxidase subunit 2 (COXII) encoded by the mitochondrial genome. However, RNA-Seq performed using mRNA extracted from the same cohort male aged mice used in the proteomic experiment revealed 18 (of 28100 identified) genes expressed differently in *Polg* mutator mice as compared with the wild-type mice and they did not overlap with proteomic data [163]. This points to the lack of direct interrelationship between proofreading defects during mtDNA replication and the development of age-related neurodegenerative pathology such as PD.

## 7. Conclusions

Convincing evidence exists that mtDNA is involved in the mitochondrial dysfunction in Parkinson’s disease. However, it still remains unclear, whether numerous mtDNA damages (oxidation, deletions, mutations, and heteroplasmy) described in the literature are the cause or consequence of PD [12,164,165]. On the one hand, the analysis of human (including postmortem brain) tissue samples demonstrating various types of mtDNA damage suggests that the recognized changes may be attributed to the final outcome of the disease. On the other hand, results of animal studies, simulating crucial stages of PD, indicate that at least mtDNA oxidation/damage, seen for example in the MPTP mouse model (see Section 7 of this review), may be referred to as one of the early events preceding the appearance of detectable changes in the levels of differentially expressed proteins. However, quantitative proteomic studies have shown that the level of mitochondrial proteins encoded by mtDNA remains basically unchanged [147,148,152]. This is a common feature found not only in the rodent but also in the MPTP-monkey model of PD. In the latter case, the proteomic analysis of postmortem brains from both *Macaca mulatta* monkeys (the rhesus monkeys) demonstrating severe parkinsonian symptoms and asymptomatic animals poorly responding to MPTP administration revealed 86 differentially expressed proteins as compared with untreated monkeys [166]. However, none of these proteins were encoded by mtDNA. The only protein related to mitochondria was the ATP synthase subunit alpha encoded by nuclear DNA [166].

Such mitochondrial resistance may be attributed to different roles of particular subunits encoded by mtDNA in the assembly and functioning of mitochondrial complexes (e.g., [167]). For example, mutations in mitochondrial genes encoding ND1 and ND3 subunits of complex I cause MELAS (mitochondrial encephalopathy, lactic acidosis, and stroke-like episodes) [168] and infantile mitochondrial encephalopathy and complex I deficiency [169], respectively. However, in the case of the ND6 subunit, the transfer of mtDNA carrying a mutation in the gene encoding this subunit to the other nuclear background exhibited a positive effect even in the absence of ND6 protein synthesis: Complex I assembly and functioning recovered [170]. Such nuclear suppression of the mitochondrial defect was also found in the case of the ND5 subunit [170]. This suggests that cross-talk between nuclear and mitochondrial genomes plays an important role in the maintenance of cell functioning. Such bidirectional cross-talk might include nuclear-encoded microRNAs influencing the expression of mitochondrial genes [171] and small mitochondrial highly transcribed RNAs (smithRNAs) regulating the expression of nuclear genes [172] and other known mechanisms (e.g., [173]). However, the applicability of such scenarios for mitochondrial dysfunction in PD requires direct experimental validation.

The role and mechanisms of nuclear suppression of mitochondrial defects clearly need better understanding as it represents a promising area for therapeutic interventions. This will be particularly interesting in the context of PD therapy.

## Figures and Tables

**Table 1:** Involvement of mtDNA in coding of components of mitochondrial complexes.

Complexes [21,22,23,24,25]	Catalytic Activity (EC)	Total Number of Subunits	Subunits Encoded by the Mitochondrial Genome
Complex I	NADH:ubiquinone reductase (EC 7.1.1.2)	44	ND1, ND2, ND3, ND4, ND4L, ND5, ND6
Complex II	Succinate dehydrogenase (EC 1.3.5.1)	4	0
Complex III	Ubiquinol—cytochrome-*c* reductase (EC 7.1.1.8)	11	1 (CYB)
Complex IV	Cytochrome *c* oxidase (EC 7.1.1.9)	13	COXI, COXII, COXIII
Complex V	ATP synthase (H^+^-transporting two-sector ATPase; EC 7.1.2.2)	14	ATP6, ATP8

**Table 2:** Mitochondrial DNA (mtDNA) mutations associated with Parkinson’s disease (PD).

Gene Encoding	Nucleotide Position in mtDNA Genome	Mutation Location	Detected Effect	References
tRNA threonine tRNA (Thr)	15888..15953	nt.15927 and nt.15928	Frequent point mutations	[95]
tRNA glutamine tRNA (Gln)	4329..4400	nt.A4336G	Loss of the HpaII site, increased frequency in PD-women The activity of complex I may be decreased	[95,96,97,98,99,100]
tRNA leucine 1 (UUA/G) tRNA (Leu)	3230..3304	nt.G3243A	point mutation, heteroplasmic state	[101]
tRNA lysine tRNA (Lys)	8295..8364	nt.A8344G	point mutation	[102]
12S ribosomal RNA (RNR1)	648..1601	nt. 956-965, nt. T1095C	5-nucleotide insertion, point mutation	[103,104]
16S RNA (RNR2)	1671..3229	nt.T2158C nt.3196	associated with reduced risk of PD heteroplasmic 16S rRNA variant	[103,105]
NADH dehydrogenase, subunit 1 (ND1)	3307..4262	nt.A3397G nt.T4216C	polymorphism	[101,106]
NADH dehydrogenase, subunit 2 (ND2)	4470..5511	nt.G5460A nt.C5178A nt.4977 p.A5T, p.A5V, p.M187T, p.M187I, p.I239M, p.I239H	point mutation point mutation common deletion amino acid substitutions	[98] [107,108] [109,110] [111,112,113]
Cytochrome *c* oxidase subunit I	5904..7445	nt.G6930A	Point mutation, causing enhanced ROS production ^a^	[114,115,116]
NADH dehydrogenase, subunit 3 (ND3)	10059..10404	nt.A10398G	point mutation Haplogroup I, J, or K had a slightly decreased risk of PD but an increased risk of PDD Protective effect for women	[99,117,118]
NADH dehydrogenase, subunit 4L (ND4L)	10470..10766	p.L77F	amino acid substitution	[111]
NADH dehydrogenase, subunit 4 (ND4)	10760..12137	nt.A11251G	point mutation associated with reduced risk of PD	[105]
NADH dehydrogenase, subunit 5 (ND5)	12337..14148	p.E145G, pE145V, p.E145D p.124-145	amino acid substitution, deletion of 30 nts	[111] [119]
12S ribosomal RNA (RNR1)	648..1601	nt. 956-965, nt. T1095C	5-nucleotide insertion point mutation	[103,104]
16S ribosomal RNA (RNR2)	1671..3229	nt.T2158C nt.3196	associated with reduced risk of PD heteroplasmic 16S rRNA variant	[105] [103]
NADH dehydrogenase, subunit 6 (ND6)	14149..14673	nt.T14487C	Point mutation causing free radical damage of cells ^b^	[120,121]
mtDNA	complete genome 16569		heteroplasmy	[122]
mtDNA	complete genome 16569 bp	Transversions G: C → T: A and T: A → G: C in point mutations	all point mutations increase with age in the frontal cortex (FCtx)	[123]

^a^ Cybrids containing mtDNA carrying the stop-codon mutation or nonsense mutation; ^b^ cybrids containing mtDNA carrying mutation in the NADH dehydrogenase subunit (see Section 5.4 of this review). PDD is Parkinson’s disease dementia.

**Table 3:** The role of mtDNA alterations in the mitochondrial dysfunction and extramitochondrial processes evaluated in PD cybrid cells.

Source of PD mtDNA	Cell Line Used to Generate Cybrids	Mitochondrial Changes	Extramitochondrial Changes	Reference
Platelets from sporadic PD patients	SHSY5Y neuroblastoma	Decreased complex I activity and increased ROS production	Increased susceptibility MPP-induced programmed cell death	[130]
PD patients with low platelet complex I activity	A549 lung adenocarcinoma	combined complex I and IV deficiencies		[131]
PD patients with reduced platelet complex I activity	NT2 teratocarcinoma cells	Decreased Complex I-IV activities	Increased LDH release, increased caspase-3 activity, increased MPP^+^-induced activation of caspase-9 and caspase-3	[132]
Platelets from sporadic PD patients	NT2 teratocarcinoma cells	Decreased Complex I activity and ATP level	Higher ROS production, Increased number of protein carbonyl groups, microtubule alteration, *α*-synuclein oligomerization	[133]
Platelets from PD patients without any nuclear DNA mutation	NT2 teratocarcinoma cells		Increased protein ubiquitination, microtubule depolymerization, and *α*-synuclein oligomerization	[134]
Platelets from PD patients	NT2 teratocarcinoma cells	Decreased mitochondrial calcium	Increased cytosolic calcium, increased calpain expression, and activation	[135]
PD patients with reduced platelet complex I activity	NT2 teratocarcinoma cells	Decreased Complex I activity, lower ATP, depolarized mitochondria, slightly increased ATP-independent proton leak	Decreased levels of PGC1α Decreased levels of SIRT1 phosphorylation Higher transcriptional activity of NF-κB	[136]
Platelets from individuals with idiopathic (sporadic) Parkinson’s disease (sPD)	SH-SY5Y Neuroblastoma cells	Insignificant trend for reduction of Complex I respiration, unaltered level of ETC subunit proteins mtDNA levels varied	mtDNA levels varied and correlated with expression of PGC-1α	[137]
Platelets from Contursi kindred PD subjects	SH-SY5Y Neuroblastoma cells	Lack of significant changes in Complex I and IV activities	Increased glutathione peroxidase	[138]
Platelets from elderly PD patients	HeLa cells	mtDNA transfer restored mitochondrial respiration of HeLa cells No significant changes were found between control and PD cybrids		[139]
Platelets from a patient with mtDNA mutation T14487C mutant	human osteosarcoma 143B cells	Overproduction of ROS causing increased oxidation of lipids and mtDNA	Increased lipid oxidation Insignificant changes in catalase and SOD	[121] [120]
Enucleated cells from patients with mtDNA mutationsA3243G in tRNA^LeuUUR^ and A8344G in tRNA^Lys^	human osteosarcoma cells (A3243G in tRNA^LeuUUR^ and A8344G in tRNA^Lys^)	both mutations showed severe deficits of complexes I, III, and IV	Increased ROS production with a parallel increase in the antioxidant enzyme activities (SOD, catalase, glutathione peroxidase)	[114]
